# Protective Effects of Fluoxetine on Decompression Sickness in Mice

**DOI:** 10.1371/journal.pone.0049069

**Published:** 2012-11-08

**Authors:** Jean-Eric Blatteau, Sandrine Barre, Aurelie Pascual, Olivier Castagna, Jacques H. Abraini, Jean-Jacques Risso, Nicolas Vallee

**Affiliations:** 1 Equipe Résidante de Recherche Subaquatique Opérationnelle. Institut de Recherche Biomédicale des Armées - Toulon, Département Environnement Opérationnel, Unité Environnements Extrêmes, Toulon, France; 2 Université de Caen Basse Normandie, Caen, France & Centre Hospitalier Affilié Université Laval Hôtel-Dieu de Lévis, Lévis, Quebec, Canada; Texas A&M University-Corpus Christi, United States of America

## Abstract

Massive bubble formation after diving can lead to decompression sickness (DCS) that can result in central nervous system disorders or even death. Bubbles alter the vascular endothelium and activate blood cells and inflammatory pathways, leading to a systemic pathophysiological process that promotes ischemic damage. Fluoxetine, a well-known antidepressant, is recognized as having anti-inflammatory properties at the systemic level, as well as in the setting of cerebral ischemia. We report a beneficial clinical effect associated with fluoxetine in experimental DCS. 91 mice were subjected to a simulated dive at 90 msw for 45 min before rapid decompression. The experimental group received 50 mg/kg of fluoxetine 18 hours before hyperbaric exposure (n = 46) while controls were not treated (n = 45). Clinical assessment took place over a period of 30 min after surfacing. At the end, blood samples were collected for blood cells counts and cytokine IL-6 detection. There were significantly fewer manifestations of DCS in the fluoxetine group than in the controls (43.5% versus 75.5%, respectively; p = 0.004). Survivors showed a better and significant neurological recovery with fluoxetine. Platelets and red cells were significantly decreased after decompression in controls but not in the treated mice. Fluoxetine reduced circulating IL-6, a relevant marker of systemic inflammation in DCS. We concluded that fluoxetine decreased the incidence of DCS and improved motor recovery, by limiting inflammation processes.

## Introduction

Scuba diving can result in the production of venous gas emboli due to the release of inert gas originally held in solution in the form of a free gas phase from peripheral tissues during decompression. When bubbles are excessively generated in blood and tissues, signs and symptoms referred to as decompression sickness (DCS) may occur [1]. Neurological damage in the spinal cord and brain underlies the most serious symptoms of DCS [2]. Even after standard treatment with hyperbaric oxygen, 20–30% of the divers affected by neurological DCS had incomplete recovery at discharge [3]. Bubble formation in blood induces activate the vascular endothelium, stimulate prothrombotic phenomena and induce inflammation: platelet and leukocyte activation have been observed, associated with elevated production of cytokines and cell adhesion stimulators [2], [4], [5]. It is now accepted that severe DCS is a systemic pathophysiological process that may induce tissue reaction that promotes ischemic damage in the spinal cord or the brain [6], [7], [8].

Recent clinical trials suggest that fluoxetine may have a neuroprotective role in stroke [9], [10]. Fluoxetine, the active compound in Prozac™, prevents the reuptake of serotonin (5-hydroxytryptamine, 5-HT) and increases the concentration of circulating serotonin [11] by inhibiting serotonin transporters (SERT) located in neurons, platelets [12] and leukocytes [13], [14], [15]. The uptake mechanism of platelet SERT regulates plasma 5-HT levels and secures stable blood flow by decreasing the possibility of platelet activation [16]. Fluoxetine is recognized as having anti-inflammatory effects by suppressing the production of IFN gamma and stimulating that of IL-10 [17]. Moreover, neuroprotective effects in the setting of cerebral ischemia are also described. Fluoxetine attenuates kainic acid-induced neuronal cell death in the mouse hippocampus and suppresses proinflammatory markers (COX-2, IL-1 beta, TNF alpha) and NF kappaB activity dose-dependently [18]. In a rat cerebral model of middle cerebral artery occlusion, fluoxetine reduced infarct volumes and improved motor impairment. The fluoxetine-treated brain was found to show marked reduction of microglia activation, neutrophil infiltration, and proinflammatory marker expressions, including NF kappaB activity [19]. Fluoxetine administered following global cerebral ischemia in mice decreased sensorimotor deficits and neuronal damage in the caudate putamen [20].

In addition to these effects in the field of cerebral ischemia, fluoxetine also has anti-inflammatory properties at the systemic level. Indeed studies with animal models and cytokine immune therapy in humans suggest that pro-inflammatory cytokines induce depressive symptomatology and it has been demonstrated that fluoxetine suppress pro-inflammatory cytokine production i.e. circulating IL-6, resulting in improvement of depressive symptoms [21], [22].

It is now believed that severe DCS is not simply a localized phenomenon but a systemic process characterized as systemic inflammatory response syndrome by Ersson *et al.* [4]. Indeed, increased levels of proinflammatory circulating cytokines especially IL-6, TNF alpha and IFN gamma have been detected in animal models of DCS, correlated with the upregulated expression of selectins in the lungs and brain [4], [23]. It has been suggested that the activation of the body’s defense system initiates a vicious cycle that leads to multiple organ failure unless the DCS is adequately treated [8].

The purpose of this research was to determine if DCS risk or severity could be reduced using fluoxetine. The secondary objective was to confirm these clinical results using biomarkers previously validated in DCS such as platelet count [24], [25], with a particular attention on the circulating level of IL-6, a relevant marker of systemic inflammation observed in DCS [4], [23].

## Materials and Methods

### Animals and Ethics Statement

All procedures involving experimental animals were in line with European Union rules (Directive 86/609) and French law (Decree 87/848). The ethics committee of IRBA approved this study. Our investigator (NV) is associated to the agreement number 83.6 delivered by the Health and Safety Directorate of our department, as stated in the French rules R.214-93, R-214-99 and R.214-102. Only C57Bl6 (Harlan Laboratories, France) males were used in this experiment in order to avoid fluctuations due to female hormone cycles. All the mice were housed in a common cage and kept–both during rest and during the experiments–on a regular day (6h00–18h00)/night (12 hours) cycle. Food (*AO3, UAR*) and water were *ad libitum* and the temperature was maintained at 22±1°C.

A total of 91 mice (6–9 weeks of age) were exposed to compressed air to induce DCS. The mice were randomly divided into two groups and numbered: 46 for the group treated with fluoxetine and 45 for the controls. Weight was similar in both groups (23.8±2.3 g for fluoxetine vs 24.3±2.3 g for controls, p = 0.304). The experimental group received a *per os* 50 mg/kg fluoxetine solution in the form of Prozac™ (Lilly laboratories, France) 18 hours before hyperbaric exposition while the control group had a similar saccharine solution (7.4 g/kg) without fluoxetine. We opted to use a high dose of fluoxetine based on previous research in a mouse model of ischemia [18], [20].

### Hyperbaric Procedure

Our hyperbaric procedure was based on previous studies using short and relatively deep no-stop dives that favour neurological symptoms of DCS [26], [27].

Batches of 18–20 freely-moving mice (9–10 per cage and per group) were subjected to the hyperbaric protocol in a 200-liter tank fitted with three ports for observation.

The compression protocol involved a rise of 10 kPa.min^−1^ up to absolute pressure of 200 kPa (equivalent depth of 10 msw), then 100 kPa.min^−1^ up to 1000 kPa (equivalent depth of 90 msw), maximal absolute pressure at which the animals were kept for 45 minutes before decompression. The decompression rate (100 kPa.sec^−1^) was automatically controlled by a computer linked to an Analogical/Digital converter (NIUSB-6211, National Instrument, USA) itself connected to a solenoid valve (Belino LR24A-SR, Switzerland) and a pressure transmitter (Pressure Transmitter 8314, Burket Fluid Control System, Germany). The program used to control decompression rate was designed on DasyLab (DasyLab National Instrument, USA) by our engineer.

Compressed air was generated using a diving compressor (Mini Verticus III, Bauer Comp, Germany) coupled to a 100-liter tank at 300 bar. The oxygen analyzer was based on a MicroFuel electrochemical cell (G18007 Teledyne Electronic Technologies/Analytical Instruments, USA).Water vapor and CO_2_ produced by the animals were respectively captured with seccagel (relative humidity: 40–60%) and soda lime (<300 ppm captured by the soda lime), respectively. Gases were mixed by an electric fan. The day-night cycle was respected throughout. The temperature inside the tank was measured using a platinum-resistance temperature probe (Pt 100, Eurotherm, France). All these variables were controlled by a dedicatedcomputer.

### Behavior and Clinical Observations

At the end of decompression, the mice were transferred to individual cages and observed during 30 minutes by a dedicated staff, blinded to treatment. The following symptoms were considered as manifestations of DCS: respiratory distress, paralysis or moving difficulties (including limping, failure to maintain balance, sideways gait, falling, difficulty righting after a fall), convulsions and death. The presence of isolated subclinical manifestation i.e. prostration (without moving difficulties after stimulation) was not considered as a specific sign of DCS. The time of onset of these symptoms were also recorded. Problems with fore or rear limbs and convulsions were classified as being due to neurological DCS.

Grip tests–motor/sensory tests adapted from Hall *et al.* [28] were used to quantify forelimb involvement 15 minutes (test session 1) and 30 minutes (test session 2) after the end of decompression: this test uses a 60 cm-long wire cord suspended at a height of 40 cm. The mouse is placed in the middle of the wire cord hanging from its front paws. Suspension time more than 30 sec is considered as a successful grip. Mice which escape by climbing up the cord during both test sessions are also considered asymptomatic and are given the highest score of 30 sec. In order to alleviate the distress experienced by the animals, all the mice were anesthetized after this period of observations.

### Blood Cell Tests

Blood tests were carried out in an automatic analyzer (ABCvet, SCIL, France) on samples taken before the dive and then again 35 minutes after surfacing. Red cells, leukocytes and platelets were counted in 20 µl samples taken from the tip of the tail and diluted in an equivalent volume of 2 mM EDTA (Sigma, France).

### Circulating IL-6 Detection

40 min after surfacing, the mice were anesthetized by intraperitoneal injection of a mixture of 16 mg/kg xylazine (Rompum® 2%, Bayer Pharma) and 100 mg/kg ketamine (Imalgene®1000, Laboratoire Rhône, France). Blood samples were collected from the inferior *vena cava* to determine the values of plasmatic IL-6 level. Blood (900 µl) was drawn up carefully into a disposable syringe with ACD (100 µl) and centrifuged immediately. At the end of the experiment, the mice were sacrificed by injecting pentobarbital (200 mg/kg ip, Sanofi Santé, France). Plasma was obtained within 30 minutes by a single centrifugation at 1 100 g and 4°C for 10 min. The supernatant was stored at −80°C until assay.

IL-6 detection was carried out with a Bioplex100 (BioradInc, CA, USA) and an immunoassay kit (Milliplex® MAP Mouse IL-6 Magnetic Bead Panel Immunoassay, Merck Millipore, USA). Samples, standards and quality controls were carried out using two duplicates per point. All standards (10000 to 16 pg/ml) and quality controls were prepared as recommended in the kit. Baseline levels of IL-6 were obtained in a group of 12 matched mice, which received no treatment and were not submitted to hyperbaric exposure.

### Statistical Analysis

For statistical processing, we used Sigmastat 3.0 (SPSS inc., Chicago, Illinois). Numerical data points were expressed as mean and standard deviation. A contingency table was used for independence and association tests, coupled with a Fisher Exact or Chi^2^ test of significance. Comparisons between multiple groups were analysed by Kruskal-Wallis test and Dunn’s test for post hoc analysis. Differences between two groups were analysed by a Mann-Whitney test, whereas matched comparisons within groups used a Wilcoxon test. A difference was considered as significant for p-values <0.05.

**Figure 1:**
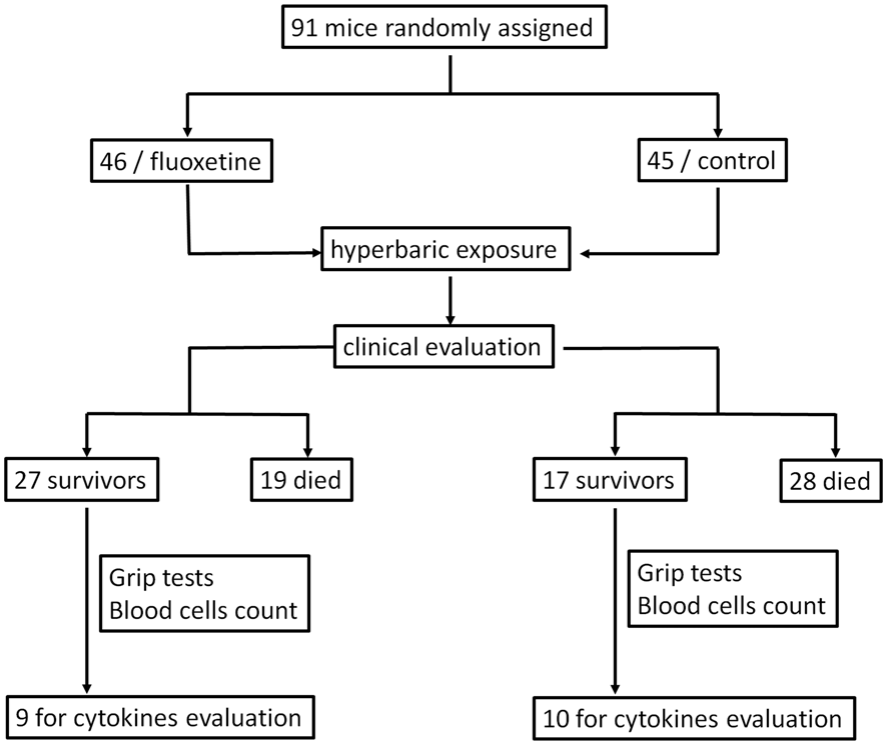
Flow chart describing the experimental design.

The experimental design is detailed in Figure 1.

## Results

### Clinical Observations

**Figure 2:**
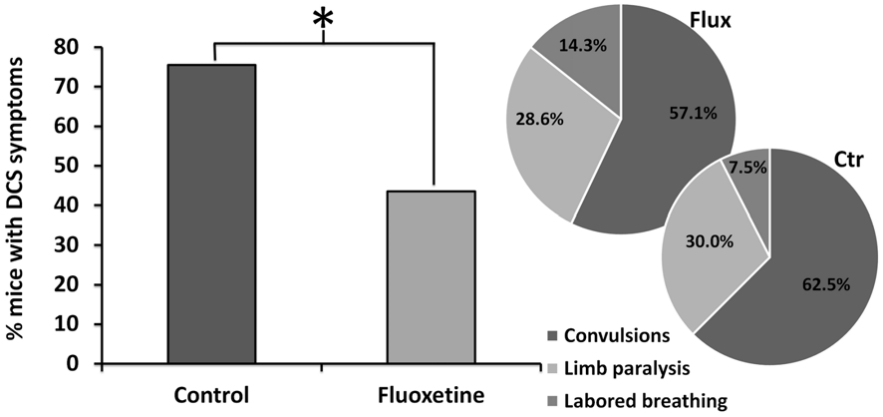
Percents of symptomatic mice suffering from decompression sickness (DCS) within 30 min after surfacing. Histogram in dark grey represents the mice treated with fluoxetine and light grey represents the controls. *denotes p<0.05 between groups. Distribution of symptoms is represented for each group.

The mice expressed mainly neurological symptoms of varying intensity including locomotor impairment (paraplegia or paraparesis) and convulsions, frequently resulting in death. All cases of neurological symptoms occurred within 14 min after surfacing. In some cases respiratory symptoms were also noted. There was significantly fewer clinical manifestations of DCS in the fluoxetine group compared with the controls (43.5% vs 75.5%, respectively, p = 0.004). The distribution of symptoms did not differ significantly between the groups (Figure 2). Death, generally preceded by convulsions, occurred more frequently in the control group, but the difference did not reach statistical significance (62.2% versus 41.3%, respectively, p = 0.074). Additional analysis found that time to onset of DCS symptoms (6.21±2.7 min for fluoxetine vs 5.43±2.9 min for controls, p = 0.404) and time to death were not significantly different (7.06±3.7 min for fluoxetine vs 5.76±2.4 min for controls, p = 0.194) between groups. Except for one case, the death occurred within 11 min after surfacing.

### Grip Tests (Figure 3)

In the subgroup of surviving mice (n = 27 in the fluoxetine group and n = 17 in the controls), motor ability of forelimbs was quantified with 2 subsequent grip tests. One mouse deceased at 19 min (in the fluoxetine group) after the first grip test and was excluded from this analysis.

**Figure 3:**
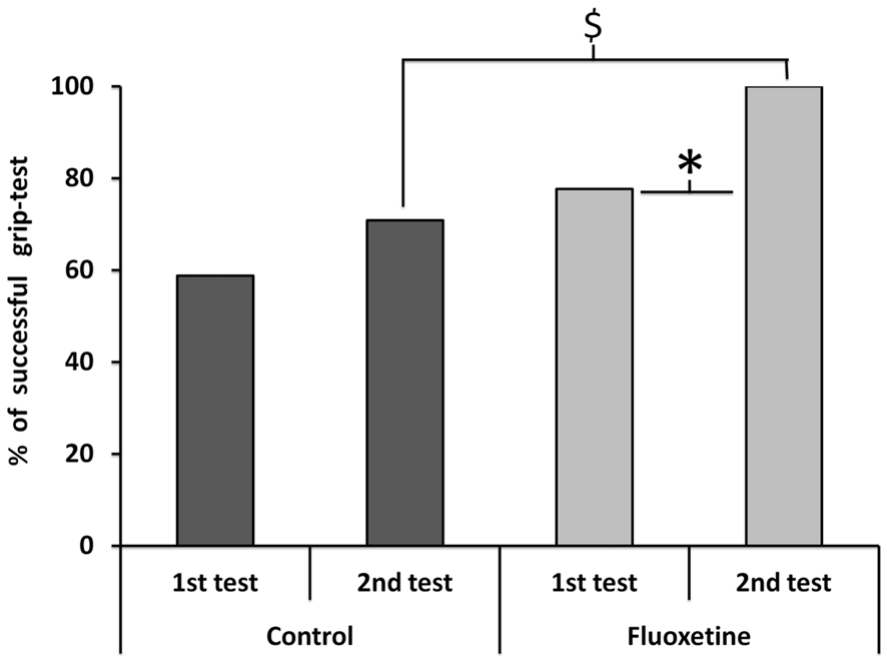
Percents of successful grip tests (suspension time ≥30 sec) in dark grey for the mice treated with fluoxetine and light grey for the controls. Grip tests were performed in each group to quantify forelimb involvement 15 and 30 min after surfacing. $ denotes p<0.05 between the groups and *denotes p<0.05 between the paired mice.

#### First grip test at 15 min

We found no significant difference between groups that passed the grip test at 15 min (77.7% for fluoxetine vs 58.8% for controls, p = 0.316).

#### Second grip test at 30 min

More mice in the fluoxetine group passed the grip test at 30 min (100% for fluoxetine vs 70.6% for controls, p = 0.006).

#### Differences between the 2 grip tests

A significant difference in performance was observed between the first and the second passed grip test in the fluoxetine group (77.7% vs 100%, respectively, p = 0.03) whereas the difference was not significant in controls (58.8 vs 70.9%, respectively, p = 0.718).

### Blood Cells (Figure 4)

#### Platelet counts

Following the dive, the platelet count was significantly reduced by −16.3±27.6% from baseline in the controls (p = 0.01) whereas the decrease by −1.77±35% was not significant in the fluoxetine group (p = 0.974).

**Figure 4:**
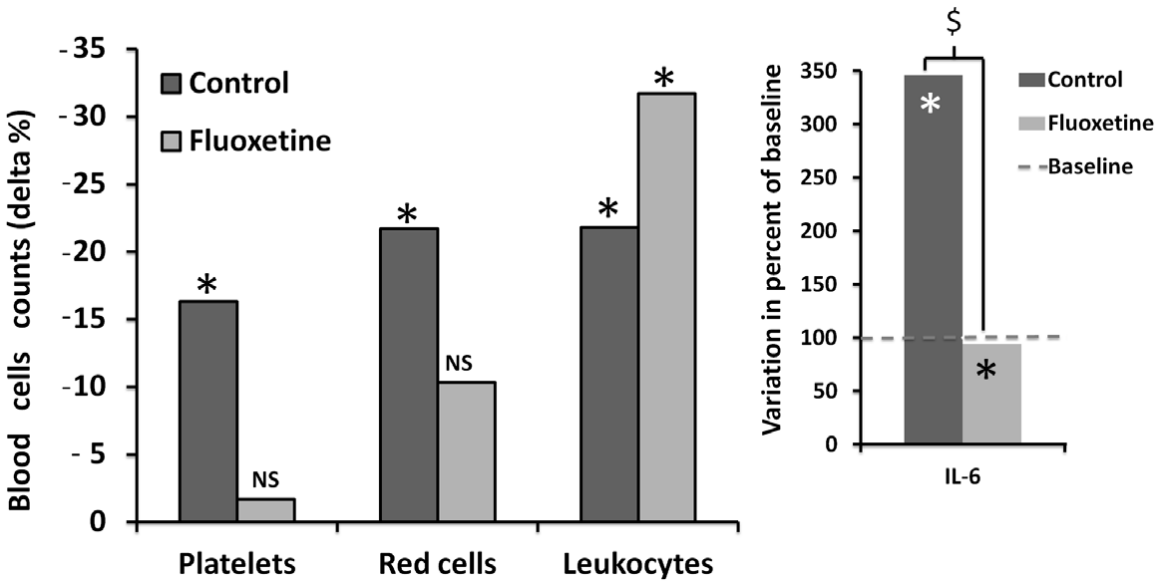
Percents of blood cells consumption after decompression from the baseline in dark grey for the mice treated with fluoxetine and light grey for the controls. *denotes a significant difference between pre- and post-decompression. On the right, changes (%) in circulating cytokine IL-6 levels after decompression from the baseline. $ denotes a significant difference between groups. *denotes p<0.05 from the baseline.

#### Leukocyte counts

Following the dive, the leukocyte count was significantly decreased from baseline by −21.8±38.8% in the controls (p = 0.025) and by −31.7±41.7% in the fluoxetine group (p<0.001), with no statistical difference between groups (p = 0.412).

#### Red cells

Following the dive, the red cell went down by −21.7±21.7% from baseline in the controls (p = 0.03) whereas the decrease by −10.2±30.3% was not significant in the fluoxetine group (p = 0.05).

### Circulating IL-6

We found a significant difference of IL-6 between the groups (p = 0.002). As shown in Figure 4, circulating levels of inflammatory cytokine IL-6 were significantly increased by 245.5±260% from baseline in the controls (n = 10) whereas IL-6 levels in the fluoxetine group (n = 9) were significantly reduced by −251.8±313% compared with the control group.

## Discussion

The aim of the present study was to investigate the effects of fluoxetine in a clinically relevant model of DCS that produces motor impairment and convulsions suggestive of spinal cord and/or brain damage, previously used in studies in mice of similar weight [26], [27]. The main finding in this study is that mice treated with fluoxetine had lower DCS incidence, as assessed both by behavioral observations and multiple biological markers.

### Effects of Fluoxetine on Motor Impairment

We observed a better neurological recovery in the fluoxetine group with an increased percent of successful suspension tests seen between the first and second grip tests. This suggests that fluoxetine could have a neuroprotective effect in neurological DCS, in line with previous studies on cerebral ischemia [19], [20].

### Effects of Fluoxetine on Blood Cells

Animal experiments strongly suggest a role for the involvement of blood components in DCS [2], [24], [29]. We found that platelet and red cell counts were significantly reduced after decompression in controls but not in treated mice. Previous animal studies reported that platelet count falls following decompression [24] and can be considered to be a relevant index for evaluating decompression stress [25]. The drop in platelet count is usually attributed to clotting activity following exposure of the collagen under bubble-damaged endothelial cells in the blood vessels [30], [31], [32], or direct interaction between bubbles and platelets [33], [34]. Our data did not reveal a drop in platelet count following decompression in treated animals, thus suggesting a beneficial role of fluoxetine in the coagulation pathway.

Antidepressants, particularly selective 5-HT reuptake inhibitors such as fluoxetine, can have a direct influence on serotonin platelet levels. 5-HT is usually a vasodilator, becoming a vasoconstrictor when the endothelium is damaged, being taken up from plasma and stored in platelet granules. Upon initiation of platelet aggregation, 5-HT is released into the blood and activates 5-HT2A receptors on the platelet membrane, which enhances the aggregation process. 5-HT *per se* is a weak activator, but dose-dependently enhances platelet activation induced by adenosine diphosphate [35]. Since Fluoxetine may inhibit platelet uptake of 5-HT and cause platelet depletion, this can inhibit 5-HT-induced platelet aggregation amplification, and therefore explain why we did not observe a drop in platelet count after decompression in the treated group.

A different interpretation can be proposed concerning red-cells. Several authors have observed phenomena of blood sludging and red-cell fragmentation/deformation following rapid decompression in animal models. The formation of red-cell aggregates appears to be associated with flow stasis [24], [36]. The red-cell count following decompression did not drop in treated animals, suggesting that blood sludging was limited in this group. Previous studies found that fluoxetine may have a positive impact on hemorheologic measures of stress-hemoconcentration by improving increased blood viscosity [37]. This effect could be mediated by fluoxetine inhibition of volume-regulated anion channels (VRAC), which are important regulators of various cell functions and has been described in neuronal and endothelial cells of the blood-brain barrier. VRAC are critically involved in volume regulation and maintain the osmotic composition of the fluid compartments in the central nervous system [38], [39].

Concerning leukocytes, we found that leukocyte count decreased after decompression, both in the control and treated groups. Experimental observations in DCS suggest that damage to the vascular endothelium by gas bubbles may provoke an inflammatory and immune response resulting in leukocyte activation [40]. The fall in leukocyte count after DCS is usually attributed to diapedesis [41], [42]. Neutrophils are the first inflammatory cells to arrive at the site in neurological tissue. Through their properties and phagocytic effect, they remove tissue debris and restore homeostasis. However, according to the degree of recruitment, neutrophils may be responsible for deleterious effects through the release of proteases and reactive oxygen species [43]. We hypothesized that fluoxetine can modulate leukocytes recruitment, with activation of anti-inflammatory pathways, limiting the deleterious role of neutrophils.

### Anti-inflammatory Effects of Fluoxetine in DCS

We found that fluoxetine reduced circulating levels IL-6. Dual function of cytokines is well established from the literature and pro-inflammatory and anti-inflammatory effects are described with IL-6, however systematic levels of IL-6 are primarily markers of disease severity, i.e. serum IL-6 often correlate with mortality in patients with septic shock [44]. Ersson *et al.* [4] found elevated serum levels of IL-6 and TNF-alpha by 6 hours after rapid decompression in rats. Bigley *et al.* [23] confirmed that rapid decompression induced the release of inflammation mediators and resulted in tissue inflammation cascades. They found that increased levels of inflammatory cytokines especially IL-6, TNF-alpha and IFN-gamma were also detected in the circulation 6 hours after decompression, but only IL-6 was still present at 24 hours.

### Animal Model of DCS

Animal experimentation is especially useful in studies that would pose unacceptable risks in human subjects. The use of a murine model is relevant in neurological DCS evaluation [26], [27], however specific problems are encountered. For example, a post-dive administration of treatment was not possible in our mouse model. Indeed the average time, from surfacing to onset of initial DCS symptoms, was very short i.e. 5 min with a high mortality rate. Moreover fluoxetine was delivered before the dive to allow the drug to reach its peak at the time of decompression. Nonetheless, we found that fluoxetine dramatically reduces the incidence of DCS and promotes motor recovery in mice. The results of this pilot study suggest that fluoxetine may reduce inflammation processes resulting from DCS, however further studies, including the assessment of inflammation markers in tissues, are needed to elucidate mechanisms of fluoxetine in DCS. It will also be necessary to determine whether the effect persists at lower doses before conducting a human trial in neurological DCS using fluoxetine as an adjunctive treatment associated with hyperbaric oxygen.
